# A Nano-MgO and Ionic Liquid-Catalyzed ‘Green’ Synthesis Protocol for the Development of Adamantyl-Imidazolo-Thiadiazoles as Anti-Tuberculosis Agents Targeting Sterol 14α-Demethylase (CYP51)

**DOI:** 10.1371/journal.pone.0139798

**Published:** 2015-10-15

**Authors:** Sebastian Anusha, Baburajeev CP, Chakrabhavi Dhananjaya Mohan, Jessin Mathai, Shobith Rangappa, Surender Mohan, Chandra, Shardul Paricharak, Lewis Mervin, Julian E. Fuchs, Mahedra M, Andreas Bender, Basappa, Kanchugarakoppal S. Rangappa

**Affiliations:** 1 Laboratory of Chemical Biology, Department of Chemistry, Bangalore University, Central College campus, Palace Road, Bangalore, 560 001, India; 2 Department of Studies in Chemistry, University of Mysore, Manasagangotri, Mysore, 570 006, India; 3 Centre for Advanced Biomedical Research and Innovation, Gulf Medical University, Ajman, United Arab Emirates; 4 Frontier Research Center for Post-genome Science and Technology, Hokkaido University, Sapporo, 060–0808, Japan; 5 Laboratory of Molecular Biology and Genetic Engineering, School of Biotechnology, Jawaharlal Nehru University, New Delhi, 110067, India; 6 Department of Studies in Physics, University of Mysore, Manasagangotri, Mysore, 570 006, India; 7 Centre for Molecular Informatics, Department of Chemistry, University of Cambridge, Lensfield Road, CB2 1EW, Cambridge, United Kingdom; 8 Division of Medicinal Chemistry, Leiden Academic Centre for Drug Research, Leiden University, P.O. Box 9502, 2300, RA Leiden, The Netherlands; University of Florida, UNITED STATES

## Abstract

In this work, we describe the ‘green’ synthesis of novel 6-(adamantan-1-yl)-2-substituted-imidazo[2,1-b][1,3,4]thiadiazoles (AITs) by ring formation reactions using 1-(adamantan-1-yl)-2-bromoethanone and 5-alkyl/aryl-2-amino1,3,4-thiadiazoles on a nano material base in ionic liquid media. Given the established activity of imidazothiadiazoles against *M*. *tuberculosis*, we next examined the anti-TB activity of AITs against the H_37_Rv strain using Alamar blue assay. Among the tested compounds 6-(adamantan-1-yl)-2-(4-methoxyphenyl)imidazo[2,1-b][1,3,4]thiadiazole (**3f**) showed potent inhibitory activity towards *M*. *tuberculosis* with an MIC value of 8.5 μM. The inhibitory effect of this molecule against *M*. *tuberculosis* was comparable to the standard drugs such as Pyrazinamide, Streptomycin, and Ciprofloxacin drugs. Mechanistically, an *in silico* analysis predicted sterol 14α-demethylase (CYP51) as the likely target and experimental activity of **3f** in this system corroborated the *in silico* target prediction. In summary, we herein report the synthesis and biological evaluation of novel AITs against *M*. *tuberculosis* that likely target CYP51 to induce their antimycobacterial activity.

## Introduction

Tuberculosis (TB) is one of the leading contagious and airborne disease caused by *Mycobacterium tuberculosis* [1, 2] and according to 2013 report of World Health Organization, TB stands second in terms of global mortality from a single infectious agent with 1.5 million death in 2013 worldwide. The conventional TB treatment comprises a cocktail of first-line drugs, namely isoniazid, pyrazinamide, ethambutol and rifampicin which are associated with lowered efficacy due to resistance development and severe adverse effects [3, 4]. The subsequent use of second-line drugs were also reported to suffer from similar problems [5–7]. Gradual increase of multidrug and extensively drug resistant (XDR-TB) mycobacterial strains demands the need of new therapeutic agents which can effectively target TB. The presence of lipid-rich cell surface on mycobacterium provides an effective therapeutic target to design anti-TB agents [8]. Researchers have rightly called adamantanyl ring as ‘lipophilic bullet’ which effectively targets mycobacterium. Evidently, hybrid obtained from the coupling of adamantylacetamide ring with 1,2,3-triazoles resulted in development of potent inhibitors against *M*. *tuberculosis* [2]. Adamantyl urea derivatives were reported to induce antimycobacterial action against *M*. *tuberculosis* [9]. SQ109, an adamantane based small molecule which is in phase-II clinical trials for the treatment of pulmonary TB [10–12]. On the other hand, Delamanid, an imidazo-oxazole based anti-tuberculosis drug was approved for the treatment of multidrug-resistant tuberculosis [13]. Thiadiazoles and imidazothiadiazoles were reported to have antitubercular activity against *M*. *tuberculosis* H_37_Rv strains [14–16]. Based on these reports, we attempted to tether the imidazo-thiadiazole nuclei to adamantyl ring in order to enhance the bioactivity profile of the newer drug-seeds. We previously developed several heterocycle based small molecules and explored the various pharmacological properties [17–25]. In the present report, we synthesized a series of novel adamantanyl-tethered imidazo-thiadiazoles for the first-time and evaluated for their inhibitory activity towards *M*. *tuberculosis*, and a subsequent mode-of-action analysis identified that they likely achieve this activity by targeting sterol 14α-demethylase (CYP51) (S1 Data).

## Results and Discussion

### Chemistry

**Fig 1:**
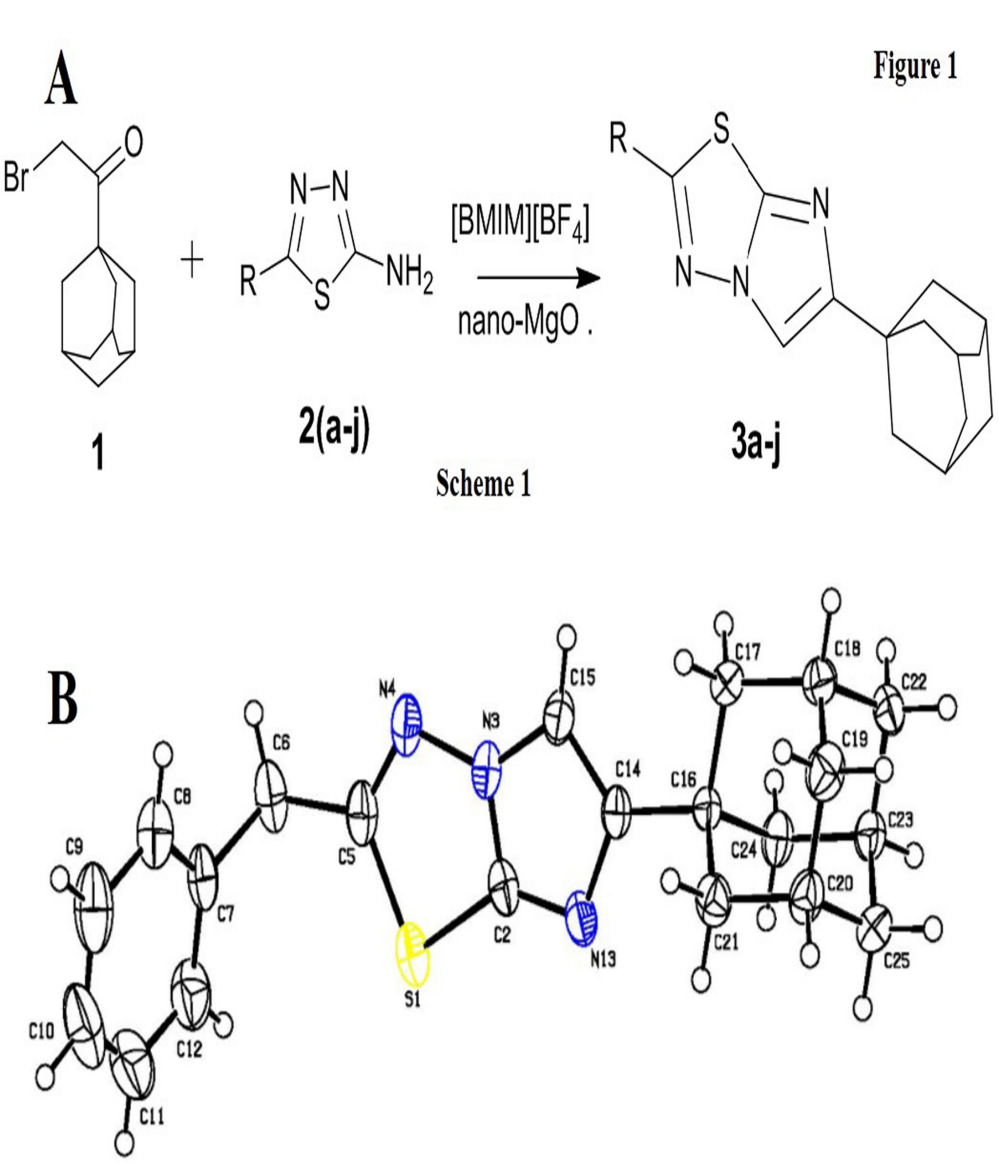
A) Schematic representation of the preparation of AITs. B) ORTEP diagram of 3b. The compound crystallizes in a triclinic system under the space group P-1, and the benzyl imidazothiadiazole moiety adopts a chair conformation with the benzyl imidazothiadiazole moiety and the phenyl ring being bridged by the carbon atom (C6) with a dihedral angle of 69.73 degrees.

The reaction between 1-(adamantan-1-yl)-2-bromoethanone and 5-substitued-2-amino-1,3,4-thiadiazoles yielded 6-(adamantan-1-yl)-2-substituted-imidazo[2,1-b][1,3,4]thiadiazoles (‘AITs’) with varying yields under different base and solvent conditions (Fig 1A, Scheme 1). Use of solvents such as ethanol, 1-butanol, N,N-dimethyl formamide resulted in poor yields. In order to overcome yield limitation, we next used various ionic liquids (ILs) in combination with a nano-catalyst, nano-MgO (Table 1) [26, 27]. ILs are molten salts, which can dramatically accelerate the rate of reactions, and have often been found to be a suitable substitute for low-boiling organic solvents in terms of toxicity, volatility and flammability [28]. In addition, ILs have more favorable ‘green’ properties, since they are reusable. In the current work, replacement of organic solvents with ILs significantly improved yields of the product to greater than 90%. In particular, [BMIM]^+^[BF_4_]^-^ and [BMPy]^+^[PF_6_]^-^ were found to be the better ILs and we have chosen [BMIM]^+^[BF_4_]^-^ for the preparation of compounds due to its solubility in water. Additionally, this method was found to be green protocol for the preparation of alkyl or aryl substitution on thiadiazole ring (Table 2). All the isolated products of the reaction were fully characterized by ^1^H NMR, LC-MS and elemental analysis. Finally, we prepared the single crystal of one of the AITs, namely **3b**, *via* slow evaporation technique. The single crystal X-ray diffraction studies of **3b** confirmed formation of the title compounds (Fig 1B).

**Table 1:** Cyclocondensation of 5-Phenyl-2-amino-1,3,4-thiadiazole (2) with 1-Adamantyl bromomethylketone (1) under various reaction conditions to form title compounds. It can be seen that using nano-MgO as a base and employing ionic liquids instead of traditional organic solvents considerably increase yields above 90%.

Entry	Reaction conditions	Reaction time (h)	Yield (%)
1	Ethanol^a^ / Na_2_CO_3_	36	37
2	1-Butanol^b^ / MgO	42	38
3	N,N-DMF^c^ /Et_3_N	30	30
4	1-Butyl-3-methyl imidazolium tetrafluoroborate^d^/ Na_2_CO_3_	25	68
5	1-Butyl-4-methyl pyridinium hexafluorophosphate^d^/ K_2_CO_3_	24	55
6	1-Butyl-3-methyl imidazolium tetrafluoroborate^d^/ Et_3_N	24	58
7	1-Butyl-4-methyl pyridinium hexafluorophosphate^d^/ MgO	14	64
8	1-Butyl-3-methyl imidazolium tetrafluoroborate^d^/ MgO	11	71
9	1-Butyl-3-methyl imidazolium tetrafluoroborate^d^/Nano MgO	3	93
10	1-Butyl-4-methyl pyridinium hexafluorophosphate^d^/ Nano MgO	3	95

^a^Reaction temperature = 80°C

^b^Reaction temperature = 95°C

^c^Reaction temperature = 120°C

^d^Reaction temperature = 60°C.

**Table 2:** Cyclocondensation of 5-alkyl/aryl-2-amino-1,3,4-thiadiazole (1a-j) with 1-adamantyl bromomethylketone to form (3a-j).

Entry	R	Product (3a-j)	Reaction time (h)	Yield (%)	Melting point (°C)	Alamar Blue Activity (μM)
**3a**	C_6_H_5_	6-(adamantan-1-yl)-2-phenylimidazo[2,1-b][1,3,4]thiadiazole	3	83	142	10.5
**3b**	CH_2_C_6_H_5_	6-(adamantan-1-yl)-2-benzylimidazo[2,1-b][1,3,4]thiadiazole	2	79	112	30.7
**3c**	4-NO_2_- C_6_H_4_	6-(adamantan-1-yl)-2-(4-nitrophenyl)imidazo[2,1-b][1,3,4]thiadiazole	3	79	163	28.7
**3d**	4-OCH_3_- C_6_H_4_CH_2_	6-(adamantan-1-yl)-2-(4-methoxybenzyl)imidazo[2,1-b][1,3,4]thiadiazole	2	86	126	32.7
**3e**	C_4_H_3_O	6-(adamantan-1-yl)-2-(furan-2-yl)imidazo[2,1-b][1,3,4]thiadiazole	2	77	131	38.2
**3f**	4-OCH_3_-C_6_H_4_	6-(adamantan-1-yl)-2-(4-methoxyphenyl)imidazo[2,1-b][1,3,4]thiadiazole	2	84	157	8.5
**3g**	4-Br-C_6_H_4_	6-(adamantan-1-yl)-2-(4-bromophenyl)imidazo[2,1-b][1,3,4]thiadiazole	3	81	161	20.0
**3h**	C_6_H_11_	6-(adamantan-1-yl)-2-cyclohexylimidazo[2,1-b][1,3,4]thiadiazole	2	74	198	36.5
**3i**	CF_3_	6-(adamantan-1-yl)-2-(trifluoromethyl)imidazo[2,1-b][1,3,4]thiadiazole	2	63	>300	12.0
**3j**	CH_3_	6-(adamantan-1-yl)-2-methylimidazo[2,1-b][1,3,4]thiadiazole	2	67	203	22.7
**Pyrazinamide**						12.5
**Streptomycin**						5.3
**Ciprofloxacin**						4.5

Equimolar mixture of **1a-j**, and **2** was dissolved in 2 ml of [BMIM]^+^[BF_4_]^-^ and the reaction was carried out in the presence of 0.1 equivalent of **Nano** MgO at 60 ^0^C.

### Anti-TB activity of novel AITs

The *in vitro* Alamar Blue assay was employed to determine the Anti-TB activity of AITs against the *M*. *tuberculosis* H_37_Rv strain as described earlier [29]. Various concentrations of AITs were added to the culture of *M*. *tuberculosis* and minimum inhibitory concentrations (MIC) of AITs were measured and the results are tabulated in Table 2. Most AITs showed inhibitory activity towards the *M*. *tuberculosis* H_37_Rv strain, suggesting that AITs possess significant anti-TB activity. Notably, Compound **3a, 3f,** and **3i** dispalyed relatively low MIC values of 10.5, 8.5 and 12.5 μM respectively when compared to the other structurally related compounds. Compounds with electron-donating phenyl, 4-methoxy phenyl, and methyl substituents attached to the imidazo-thiadiazole scaffold were favorable for activity against *M*. *tuberculosis*.

### *In silico* molecular interactions of AITs towards sterol 14α-demethylase

**Fig 2:**
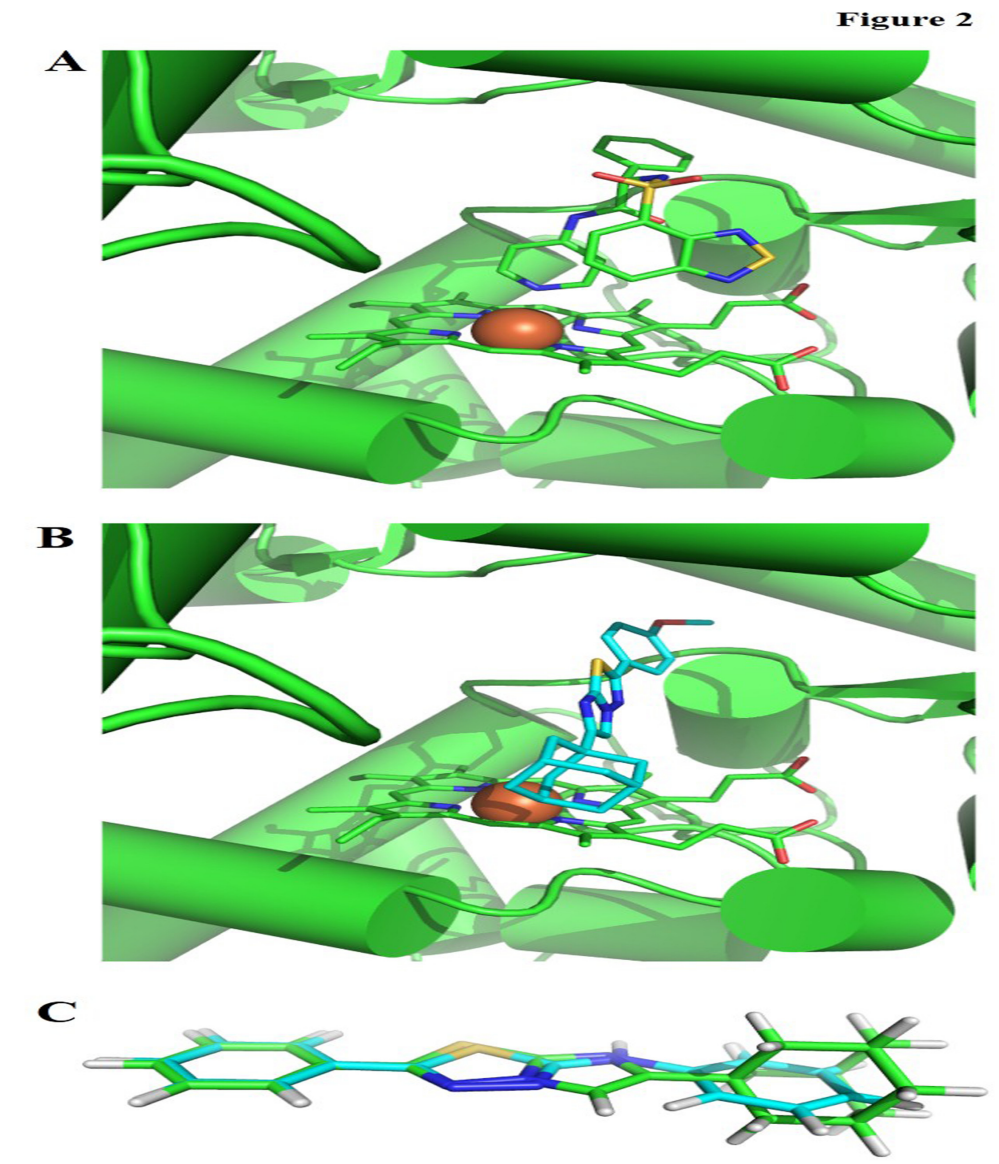
Computational binding mode analysis of AITs and *M*. *tubercolosis* CYP51. A) X-ray structure of CYP51 (green cartoon representation with heme cofactor as sticks with bound iron as brown sphere) in complex with a small molecule inhibitor (PDB: 2CIB). B) Similar interactions and an analogous three-dimensional arrangement are shown for compound **3f** of the AITs (cyan sticks). C) Overlay of the parent 1,3,4-thiadiazole of Oruc et al [Oruc04] with compound **3a** of the AITs. Positioning of ring centers, exit vectors, and overall shape are very similar, thereby plausibly explaining a similar bioactivity profile.

As sterol 14α-demethylase (CYP51) is known to process a variety of sterols and as a drug target in *M*. *tubercolosis* [30, 31], we attempted to rationalize the anti-TB activity of the AITs synthesized in this work on a structural basis. Therefore, we docked all AITs to the X-ray structure of *M*. *tubercolosis* CYP51 in complex with a small molecule inhibitor (PDB: 2CIB) [32] using MOE default settings (Fig 2A) [33] and visualized predicted protein-ligand interactions with Pymol [34]. It was found that the imidazo-thiadiazole scaffold of **3f** likely interacts with the heme co-factor of CYP51 (see Fig 2B). Furthermore, the hydrophobic moieties are positioned in similar positions to the ring centers found in the co-crystallized ligand. Based on this analysis, CYP51 appeared to be a plausible target for AITs on a structure-based level. Further, in order to analyze the similarities in binding mode between AITs, we superposed the ligand in the co-crystal used for docking with compound **3a** using MOE's flexible alignment module and default settings [33]. We found an almost perfect shape overlap of the lowest energy conformations of AITs with all hydrophobic centers coinciding with the co-cyrstallized ligand (see Fig 2C). Therefore, the AITs presented in this work could be considered a continuation of the 1,3,4-thiadizole series presented earlier by Oruc et al. [16] including an isosteric replacement of the core ring fragment.

### *In vitro* anti-microbial activity of AITs against fungal strains that express 14α-demethylase (CYP51)

Our *in vitro* and *in silico* studies revealed that AITs showed good anti-TB activity at the low micro molar concentrations, and by plausibly targeting sterol 14α-demethylase (CYP51). In order to find further experimental support for the mode-of-action analysis of the AITs presented here, the CM237 and akuB strains *of A*. *fumigatus* that express CYP51 were selected for the next step. We further investigated the effect of AITs at various concentrations against both the fungal strains by broth microdilution method as reported previously [35]. MICs were determined visually in duplicate and recorded after 48 h. Results are summarized in Table 3. Interestingly, the most active compounds against *M*. *tuberculosis*, namely **3a, 3f,** and **3i** also exhibited significant inhibitory effect against the tested *A*. *fumigates* strains. These results lend further support–though not definite proof to their plausible mode-of-action by targeting sterol 14α-demethylase (CYP51).

**Table 3:** MIC values obtained from the lead AIT compounds against *A*. *fumigates*, which expresses CYP51. Given the activity of compounds in this system this finding corroborates CYP51 as a plausible target of the AIT series.

*Aspergillus fumigatus strain*	Compound 3a MIC (μg/ml)	Compound 3f MIC (μg/ml)	Compound 3i MIC (μg/ml)
WT 237	16	16	16
WT akuB	8	8	8

## Materials and Methods

All solvents used were of analytical grade and reagents used were purchased from Sigma-Aldrich chemicals. All IR spectra were obtained in a KBr disc on a Shimadzu FT-IR 157 Spectrometer. ^1^H and ^13^C NMR spectra were recorded on a Bruker WH-200 (400 MHz) in CDCl_3_ or DMSO-d_6_ as solvent, using tetramethylsilane (TMS) as an internal standard and chemical shifts are expressed as ppm. Mass spectra were determined on a Shimanzu LC-MS. The elemental analyses were carried out using an Elemental Vario Cube CHNS rapid Analyzer. The progress of the reaction was monitored by TLC pre-coated silica gel G plates.

### Typical procedure for the synthesis of AITs

A mixture of 5-alkyl/aryl-2-amino1,3,4-thiadiazole (0.01 mol), 1-adamantyl bromomethylketone (0.01 mol) and nano magnesium oxide (0.001 mol) in 2 ml of 1-Butyl-3-methylimidazolium tetrafluoroborate [BMIM]^+^[BF_4_]^-^ was stirred at 60°C for the appropriate time (Table 2). After completion of the reaction, as determined by TLC, the reaction mixture was cooled down and then quenched into ice water. The product was extracted from the water layer by 3×5 mL diethyl ether, dried with magnesium sulfate, filtered, and concentrated *in vacuo*. The crude product was purified by chromatography employing a column of 30 mm diameter using 60–120 silica gel and hexane/ethyl acetate (80:20) as mobile phase. All new compounds exhibited spectral properties consistent with the assigned structures (S2 Data).

***6-(Adamantan-1-yl)-2-phenylimidazo[2*,*1-b][1*,*3*,*4]thiadiazole (3a)*:** ^1^H NMR (400 MHz, CDCl_3_) δ: 8.0 (s, 1H), 7.8 (m, 2H), 7.6 (m, 2H), 7.4 (m, 1H), 2.1 (m, 6H), 1.9 (m, 3H), 1.7 (m,6H); ^13^C NMR (CDCl_3_): 175.73, 134.92, 133.26, 132.17,129.39, 128.09, 127.56, 123.43, 45.59, 42.75, 38.19, 28.48; LCMS (MM:ES+APCI) 336.3 (M+H)^+^; Anal.Calcd for C_20_H_21_N_3_S: C 71.61; H 6.31; N 12.53. Found: C, 71.43; H, 6.47; N, 12.66.

***6-(Adamantan-1-yl)-2-benzylimidazo[2*,*1-b][1*,*3*,*4]thiadiazole (3b)*:** ^1^H NMR (400 MHz, CDCl_3_) δ: 8.0 (s, 1H), 7.4 (m, 2H), 7.3 (m, 3H), 4.4 (s, 2H), 1.7–2.2 (m,15H); ^13^C NMR (CDCl_3_); 161.09, 135.82, 134.73, 129.04, 128.23, 125.56, 123.65, 45.56, 42.78, 38.65, 38.32, 28.66; LCMS (MM:ES+APCI) 350.3(M+H)^+^; Anal. Calcd for C_21_H_23_N_3_: C, 72.17; H, 6.63; N, 12.02. Found: C, 71.98; H, 6.78; N 12.24.

***6-(Adamantan-1-yl)-2-(4-nitrophenyl)imidazo[2*,*1-b][1*,*3*,*4]thiadiazole (3c)*:** ^1^H NMR (400 MHz, CDCl_3_) δ: 8.1 (d, 2H), 8.0 (s, 1H), 7.9 (d, 2H), 2.1(m,6H),1.9(m, 3H), 1.7(m,6H); ^13^C NMR (CDCl_3_); 175.64, 148.97, 139.92, 135.33, 134.83, 128.65, 123.34, 121.78, 45.56, 42.78, 38.12, 28.55; LCMS (MM:ES+APCI) 381.2 (M+H)^+^; Anal. Calcd for C_20_H_20_N_4_O_2_S: C, 63.14; H, 5.30; N, 14.73. Found: C, 63.18; H, 5.88; N, 13.89.

***6-(Adamantan-1-yl)-2-(4-methoxybenzyl)imidazo[2*,*1-b][1*,*3*,*4]thiadiazole (3d)*:** ^1^H NMR (400 MHz, CDCl_3_) δ: 8.0 (s, 1H), 7.9 (d, 2H), 7.6 (d, 2H), 4.3 (s, 3H), 4.0 (s, 2H), 2.1 (m, 6H),1.9(m, 3H), 1.7(m, 6H); ^13^C NMR (CDCl_3_); 161.06, 159.43, 135.92, 134.83, 130.33, 128.23, 123.45, 114.45, 55.89, 45.56, 42.78, 38.22, 28.51; LCMS (MM:ES+APCI) 380.2 (M+H)^+^; Anal. Calcd for C_22_H_25_N_3_OS: C, 69.62; H, 6.64; N, 11.07. Found: C, 69.48; H, 7.03; N, 11.86.

***6-(Adamantan-1-yl)-2-(furan-2-yl)imidazo[2*,*1-b][1*,*3*,*4]thiadiazole (3e)*:** ^1^H NMR (400 MHz, CDCl_3_) δ: 8.1 (d, 1H), 8.00 (s, 1H), 7.4 (d, 2H), 6.8 (t, 1H), 2.1 (m, 6H), 1.9 (m, 3H), 1.7 (m, 6H); ^13^C NMR (CDCl_3_); 146.29, 142.97, 135.92, 134.83, 123.65, 107.45, 105.66, 45.56, 42.78, 38.22, 28.51; LCMS (MM:ES+APCI) 326.3 (M+H)^+^; Anal. Calcd for C_18_H_19_N_3_OS: C, 66.43; H, 5.88; N, 12.91. Found: C, 65.83; H, 6.05; N, 13,44.

***6-(Adamantan-1-yl)-2-(4-methoxyphenyl)imidazo[2*,*1-b][1*,*3*,*4]thiadiazole (3f)*:** ^1^H NMR (400 MHz, CDCl_3_) δ: 8.2 (d, J = 8.4 Hz, 1H), 8.00 (s, 1H), 7.8 (d, 2H), 4.4 (s, 3H), 2.1(m,6H), 1.9–1.7(m,9H); ^13^C NMR (CDCl_3_); 175.11, 160.63, 135.99, 134.83, 128.79, 125.32, 123.65, 114.45, 55.79, 45.56, 42.78, 38.22, 28.51; LCMS (MM:ES+APCI) 366.6 (M+H)^+^; Anal. Calcd for C_21_H_23_N_3_OS: C, 69.01; H, 6.34; N, 11.50. Found: C, 68.33; H, 5.89; N,11.10.

***6-(Adamantan-1-yl)-2-(4-bromophenyl)imidazo[2*,*1-b][1*,*3*,*4]thiadiazole (3g)*:** ^1^H NMR (400 MHz, CDCl_3_) δ: 8.3 (d, 2H), 8.1 (d, 2H), 8.0 (s, 1H), 2.0 (m, 6H),1.8 (m, 3H), 1.6 (m, 6H); ^13^C NMR (CDCl_3_); 175.14, 135.92, 134.87, 132.36, 129.56, 123.65, 45.57, 42.78, 38.29, 28.55; LCMS (MM:ES+APCI) 415.1 (M+H)^+^; Anal. Calcd for C_20_H_20_BrN_3_S: C, 57.97; H, 4.87; N, 10.14. Found: C, 57.01; H, 4,22; N, 9.56.

***6-(Adamantan-1-yl)-2-cyclohexylimidazo[2*,*1-b][1*,*3*,*4]thiadiazole (3h)*:** ^1^H NMR (400 MHz, CDCl_3_) δ: 8.0 (s, 1H), 2.7 (m, 2H), 2.1–1.8 (m, 15H), 1.7 (m, 6H), 1.5–1.4 (m, 3H); ^13^C NMR (CDCl_3_); 135.89, 134.88, 123.67, 45.61, 42.83, 38.29, 33.27, 28.58, 27.95, 25.46; LCMS (MM:ES+APCI) 342.3 (M+H)^+^; Anal. Calcd for C_20_H_27_N_3_S: C, 70.34; H, 7.97; N, 12.30. Found: C, 70.87; H, 8.54; N, 11.67.

***6-(Adamantan-1-yl)-2-(trifluoromethyl)imidazo[2*,*1-b][1*,*3*,*4]thiadiazole (3i)*:** ^1^H NMR (400 MHz, CDCl_3_) δ: 8.0 (s, 1H), 2.1 (m,6H),1.9 (m, 3H), 1.7 (m,6H); ^13^C NMR (CDCl_3_); 135.92, 134.88, 123.55, 45.54, 42.72, 38.21, 28.54, 17.34; LCMS (MM:ES+APCI) 328.4(M+H)^+^; Anal. Calcd for C_15_H_16_F_3_N_3_S: C, 55.03; H, 4.93; N, 12.84. Found: C, 55.37; H, 4.44; N, 12.13.

***6-(Adamantan-1-yl)-2-methylimidazo[2*,*1-b][1*,*3*,*4]thiadiazole (3j)*:** ^1^H NMR (400 MHz, CDCl_3_) δ: 8.0 (s, 1H), 2.8 (s, 3H), 2.1 (m,6H), 1.9 (m, 3H), 1.7 (m,6H); ^13^C NMR (CDCl_3_); 157.89, 135.92, 134.83, 123.65, 118.45, 45.56, 42.78, 38.22, 28.51; LCMS (MM:ES+APCI) 274.4 (M+H)^+^; Anal. Calcd for C_18_H_14_FNO: C, 65.90; H, 7.00; N, 15.37. Found: C, 66.09; H, 7.65; N, 15.89.

### X-ray crystal structure determination of (3b)

A single crystal of compound **3b** with dimensions of 0.30 × 0.25 × 0.20 mm was chosen for X-ray diffraction studies. The data were collected on a Bruker SMART APEX II X-ray diffractometer with Cu Kα radiation. Raw data was processed and reduced by using APEX2 and SAINT [36, 37]. The crystal structure was solved by direct methods using SHELXS-97 [38]. All non-hydrogen atoms were revealed in the first Fourier map itself. Anisotropic refinement of non-hydrogen atoms was started at this stage. Subsequent refinements were carried out with anisotropic thermal parameters for non-hydrogen atoms and isotropic temperature factors for the hydrogen atoms which were placed at chemically acceptable positions. Full-matrix least squares refinement was carried out using SHELXL-97 [39] with a final residual value of R1 = 0.079. The thermal ellipsoid plot [40] of the molecule at 50% probability is represented in Fig 1B. The details of crystallographic information have been deposited at the CCDC with number 1056579.

The crystal structure analysis showed that the compound **3b** crystallizes in a triclinic system under the space group *P-1*, with cell parameters a = 6.3060(5) Å, *b* = 10.4279(7) Å, *c* = 14.1099(10) Å, *α* = 81.101(2)°, *β* = 79.845(2)°, *γ* = 82.918(2)° and *Z* = 2. The benzyl imidazothiadiazole moiety adopts a chair conformation with puckering parameters Q = 0.622(4) Å and φ = 201(19)° [31] and the maximum deviation found on the puckered atom at C14 is 0.255(3) Å. The benzyl imidazothiadiazole moiety and the phenyl ring are bridged by the carbon atom (C6) with a dihedral angle of 69.73(5)°. The structure does not contain any classical hydrogen bonds.

### Anti-tubercular activity assay

All the novel AITs were screened for anti-tubercular activity against *M*. *tuberculosis* H_37_Rv strain (ATCC 27294) using a microplate Alamar Blue assay (MABA) as described previously [29, 31]. Briefly, 200 μl of sterile deionized water was added to all outer-perimeter wells of sterile 96-well plates to minimize evaporation of the medium in the test wells during incubation. The wells in rows B to G in columns 3 to 11 received 100 μl of Middlebrook 7H9 broth. 100 μl of 2X drug solutions were added to the wells in rows B to G in columns 2. 100 μl was transferred from column 2 to column 3, and the contents of the wells were mixed well. Identical serial 1:2 dilutions were continued through column 10, and 100 μl of excess medium was discarded from the wells in column 10 in order to get the final concentration of 0.2 μg/ml. 100 μl of *M*. *tuberculosis* inoculum was added to the wells in rows B to G in columns 2 to 11 (yielding a final volume of 200 μl per well). Thus, the wells in column 11 served as drug-free control. The plate was sealed with parafilm and incubated at 37°C for 5 days. Thereafter, 50 μl of freshly prepared 1:1 mixture of Alamar Blue reagent and 10% tween-80 was added to each well and incubated for 24 h. Appearance of blue color was interpreted as no bacterial growth, and pink color was used as indicator of bacterial growth. The inhibitory activity of AITs against *M*. *tuberculosis* was expressed as the minimum inhibitory concentration (MIC) in μM. MIC was defined as lowest drug concentration which prevented the color change from blue to pink. Pyrazinamide, streptomycin, and ciprofloxacin were used as a positive controls.

### Molecular docking analysis

*In silico* molecular docking was performed based on the X-ray structure of *M*. *tubercolosis* CYP51 in complex with a small molecule inhibitor (PDB: 2CIB) [32]. The ligand structures were energy-minimized and protonated using the MOE platform [33]. The protein was prepared for docking using protonate3D [41]. Afterwards, we docked all AITs to the X-ray structure of using MOE's default settings for flexible docking. This includes an initial placement using Triangle Matcher, primary scoring via London dG and a forcefield refinement of 30 poses followed by a re-scoring step using GBVI/WSA dG. We visualized predicted protein-ligand interactions with pymol [34].

### Anti-microbial activity

The CM237 and akuB strains *of Aspergillus fumigatus*, that expresses CYP51, were used in this work. Fungal strains were grown in Potato Dextrose Agar (PDA, Becton Dickinson, Madrid, Spain) at 37°C. After three days of growth a suspension of spores was prepared by harvesting the surface of the culture with phosphate buffered saline (PBS) plus 0.01% Tween 20. Inoculum size was then adjusted using a hemocytometer chamber according to needs. Stock solutions of each compound were first dissolved in chloroform and subjected to serial dilution to obtain the concentrations of 64, 32, 16, 8, 4, 2, 1, 0.5, 0.25 and 0.125 μg/ml. Each compound was tested between 64 μg/ml and 0.125 μg/ml. Compounds susceptibility was determined by broth microdilution (BMD) using RPMI 2% glucose. MICs were determined visually and recorded after 48 h. Amphotericin B and chloroform were used as controls of the study.

## Conclusion

In this work, we synthesized novel AITs using 1-adamantyl bromomethylketone and 5-alkyl/aryl-2-amino1,3,4-thiadiazoles employing nano-MgO in ionic liquid media. We experimentally confirmed the anti-TB activity of all AITs against *M*. *tuberculosis* H_37_Rv strain with MICs in the low micromolar range. Subsequent docking studies revealed sterol 14α-demethylase (CYP51) as a plausible target, and subsequent activity determination against fungal strains that express sterol 14α-demethylase (CYP51) corroborated this hypothesis. In summary, we herein report the synthesis and anti-TB activity of novel AITs that likely target sterol 14α-demethylase (CYP51) to induce their antimycobacterial activity.

## Supporting Information

S1 Data Graphical abstract which provides the overview of the present work. (DOCX)

S2 Data Scanned spectral images and structural analysis of novel adamantyl-imidazolo-thiadiazole derivatives. (DOCX)
