# Supplementary material for: A Nano-MgO and Ionic Liquid-Catalyzed ‘Green’ Synthesis Protocol for the Development of Adamantyl-Imidazolo-Thiadiazoles as Anti-Tuberculosis Agents Targeting Sterol 14α-Demethylase (CYP51)
Source: PLoS One. 2015 Oct 15;10(10):e0139798. doi: 10.1371/journal.pone.0139798 (PMC4607480; doi:10.1371/journal.pone.0139798)
Supplement: S1 Data — (DOCX) [file pone.0139798.s001.docx]

**Nano-MgO and ionic liquid catalyzed green protocol for the development of adamantyl-imidazolo-thiadiazoles as anti-tuberculosis agent that target sterol 14α-demethylase.**

Sebastian Anusha, Baburajeev CP, Shobith Rangappa, Surender Mohan, Chandra, Shardul Paricharak, Lewis Mervin, Julian E. Fuchs, Mahedra M, Andreas Bender, Basappa, Kanchugarakoppal S. Rangappa.
