# Supplementary material for: A Nano-MgO and Ionic Liquid-Catalyzed ‘Green’ Synthesis Protocol for the Development of Adamantyl-Imidazolo-Thiadiazoles as Anti-Tuberculosis Agents Targeting Sterol 14α-Demethylase (CYP51)
Source: PLoS One. 2015 Oct 15;10(10):e0139798. doi: 10.1371/journal.pone.0139798 (PMC4607480; doi:10.1371/journal.pone.0139798)

3a) 6-(adamantan-1-yl)-2-phenylimidazo[2,1-b][1,3,4]thiadiazole: 1HNMR (400 MHz, CDCl3) 8.0 (s, 1H), 7.8 (m, 2H), 7.6 (m, 2H), 7.4 (m, 1H), , 2.1(m,6H),1.9(m, 3H), 1.7(m,6H); LCMS (MM:ES+APCI) 336.3 (M+H)^+^ ; Anal.Calcd for C_20_H_21_N_3_S : C 71.61; H 6.31; N 12.53. Found: C, 71.43; H, 6.47; N, 12.66.


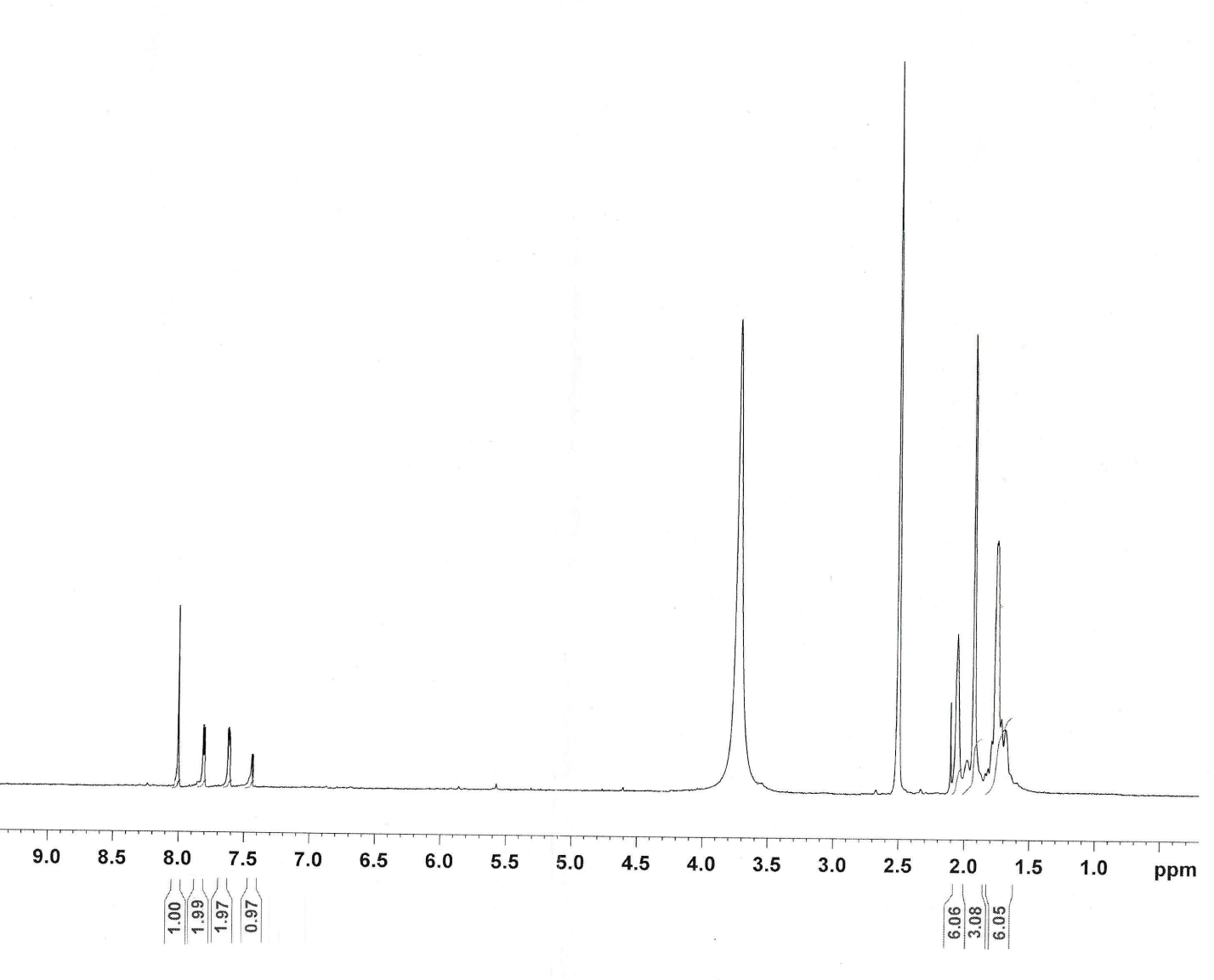


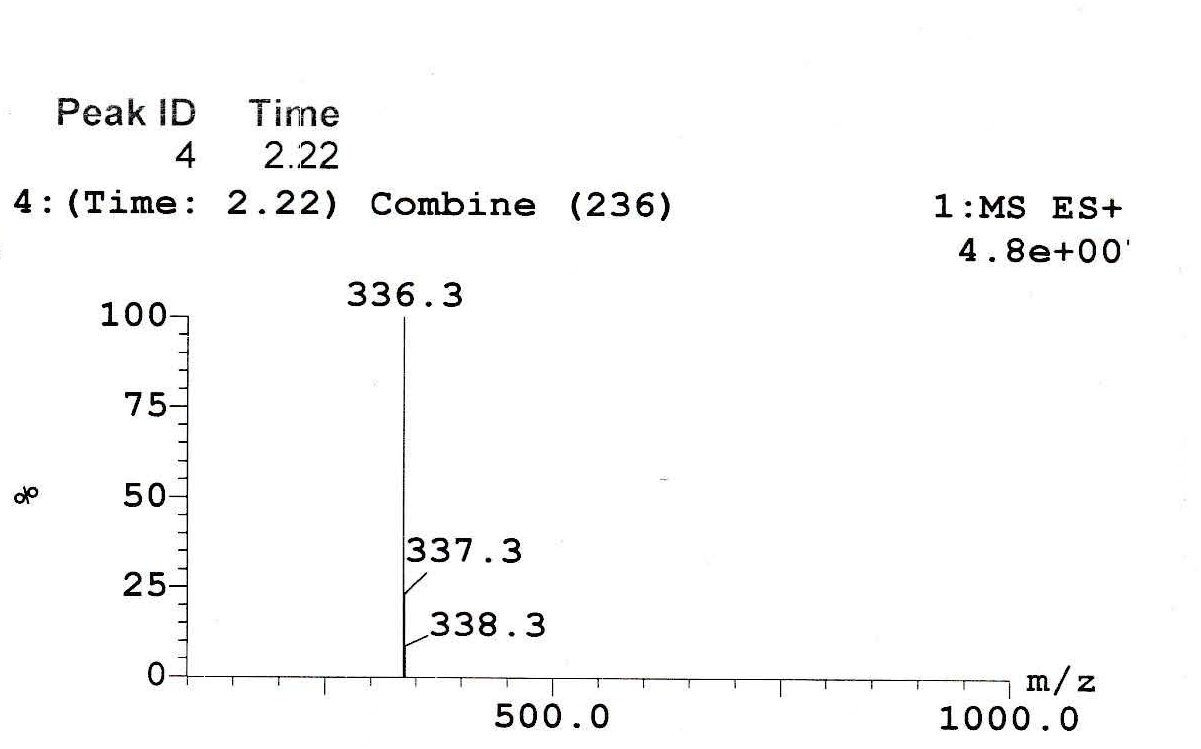

3b) 6-(adamantan-1-yl)-2-benzylimidazo[2,1-b][1,3,4]thiadiazole: 1HNMR (400 MHz, CDCl3) 8.0 (s, 1H), 7.4 (m, 2H), 7.3 (m, 3H), 4.4 (s, 2H), 1.7- 2.2(m,15H); LCMS (MM:ES+APCI) 350.3(M+H)^+^ ; Anal.Calcd for C_21_H_23_N_3_S : C 72.17; H 6.63; N 12.02. Found: C 71.98; 6.78 ; N 12.24.


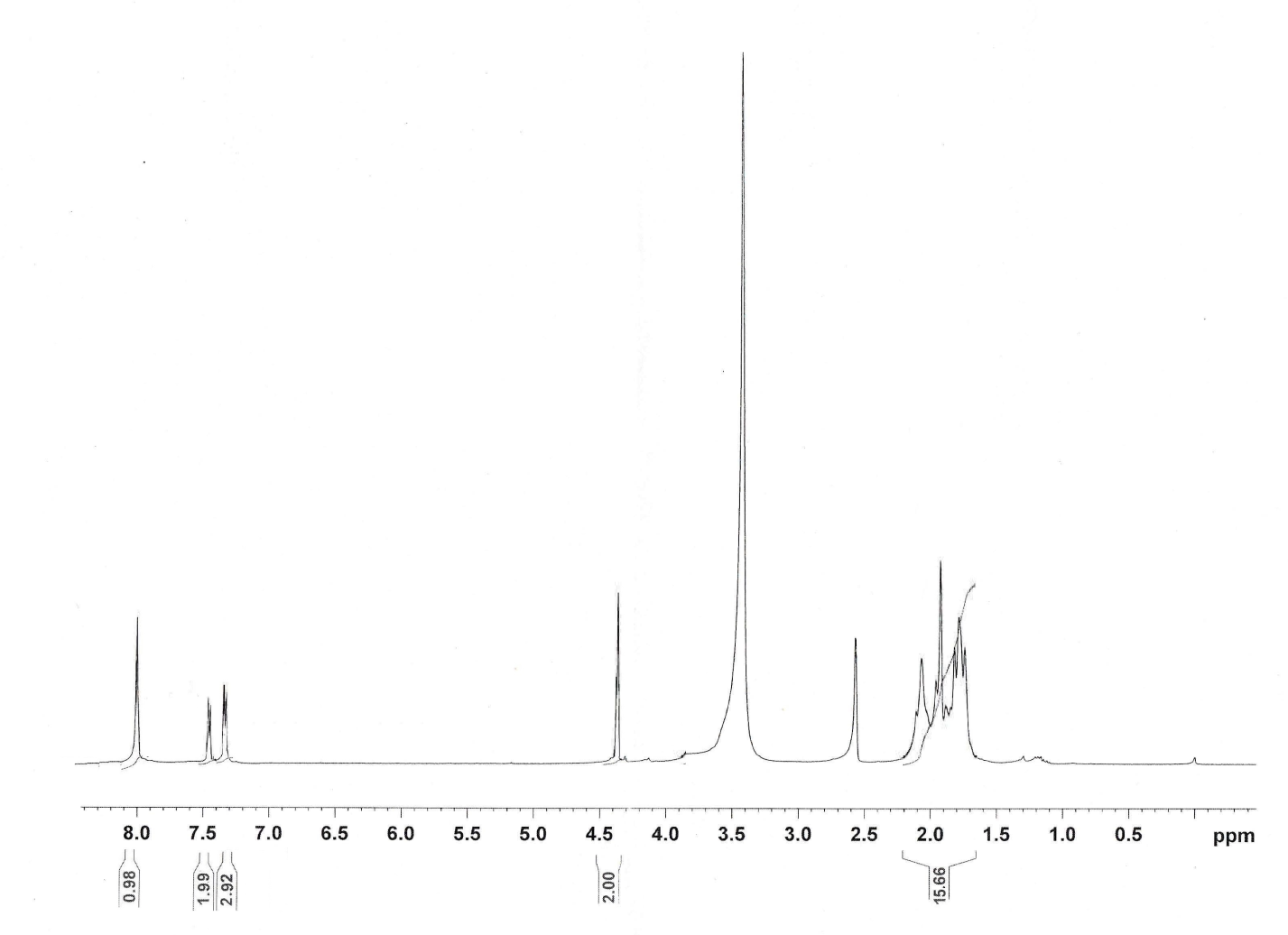


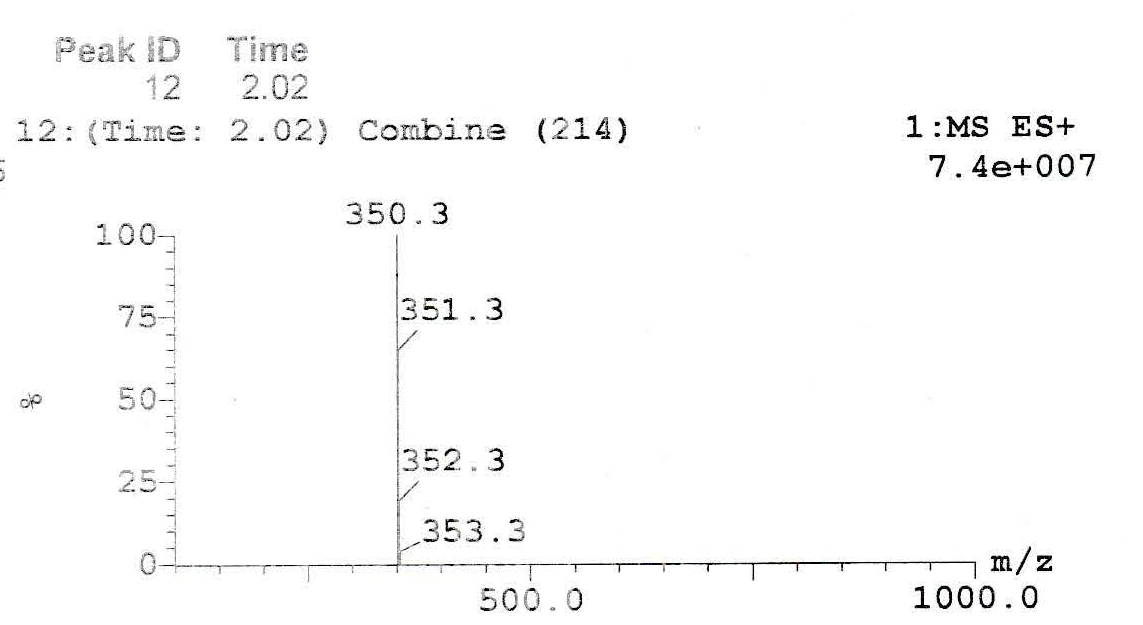

3c) 6-(adamantan-1-yl)-2-(4-nitrophenyl)imidazo[2,1-b][1,3,4]thiadiazole: 1HNMR (400 MHz, CDCl3) 8.1 (d, 2H), 8.0 (s, 1H), 7.9 (d, 2H), 2.1(m,6H),1.9(m, 3H), 1.7(m,6H); LCMS (MM:ES+APCI) 381.2(M+H)^+^ ; Anal.Calcd for C_20_H_20_N_4_O_2_S: C, 63.14; H, 5.30; N, 14.73. Found: C, 63.18; H, 5.88; N, 13.89.


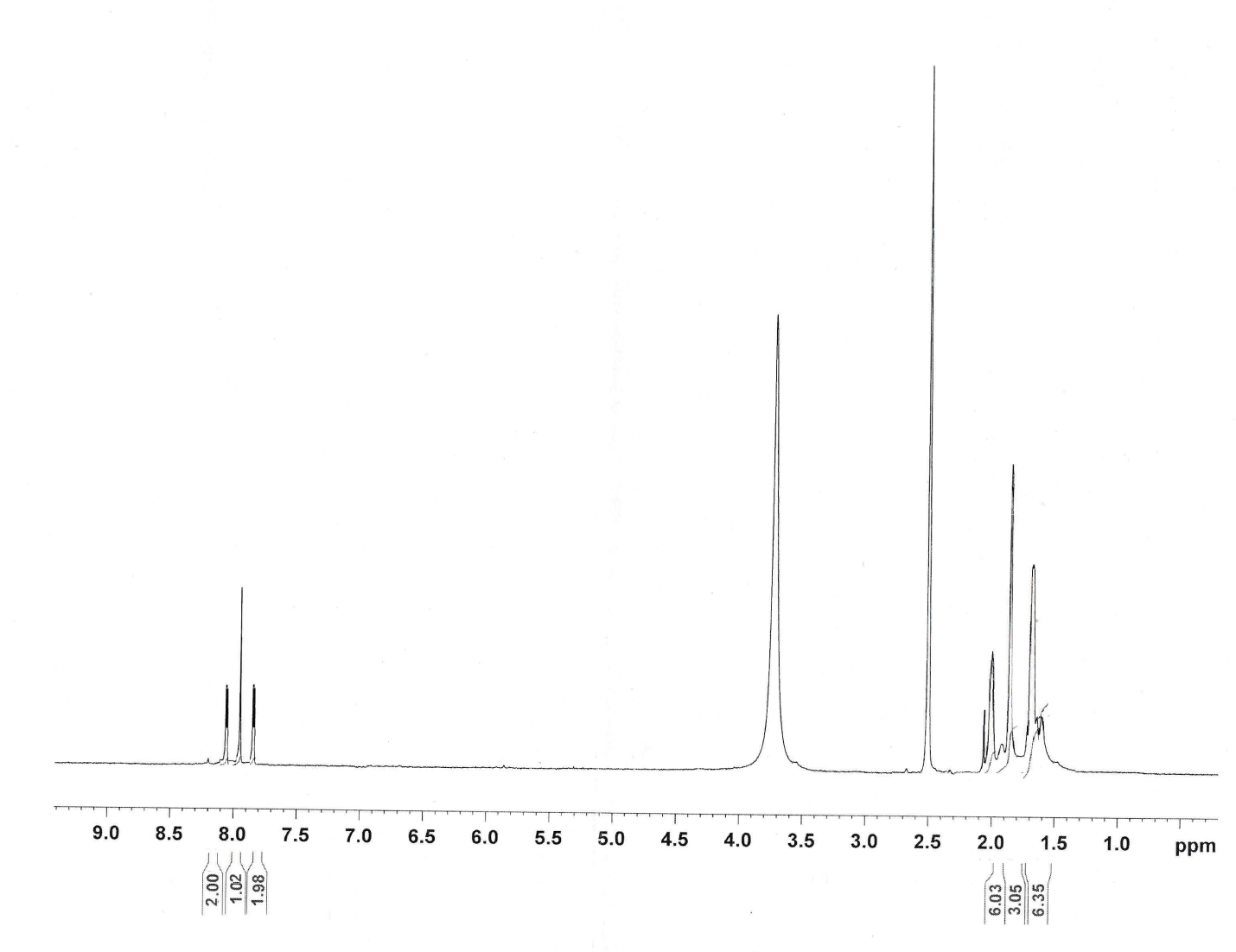


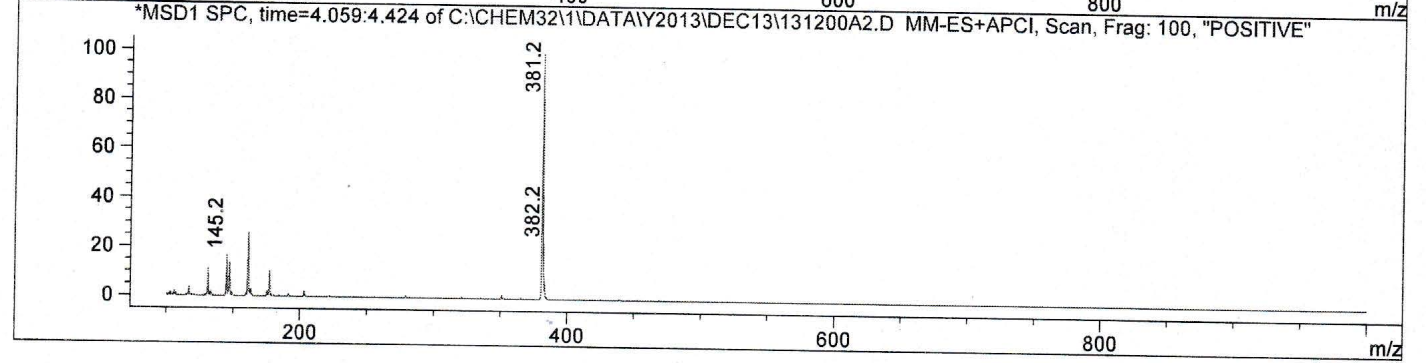

3d) 6-(adamantan-1-yl)-2-(4-methoxybenzyl)imidazo[2,1-b][1,3,4]thiadiazole: 1HNMR (400 MHz, CDCl3) 8.0 (s, 1H), 7.9 (d, 2H), 7.6 (d, 2H), 4.3 (s, 3H), 4.0 (s, 2H), 2.1(m,6H),1.9(m, 3H), 1.7(m,6H); LCMS (MM:ES+APCI) 380.2(M+H)^+^ ; Anal.Calcd for C_22_H_25_N_3_OS : C 69.62; H 6.64; N 11.07. Found: C 69.48; H 7.03 ; N 11.86.


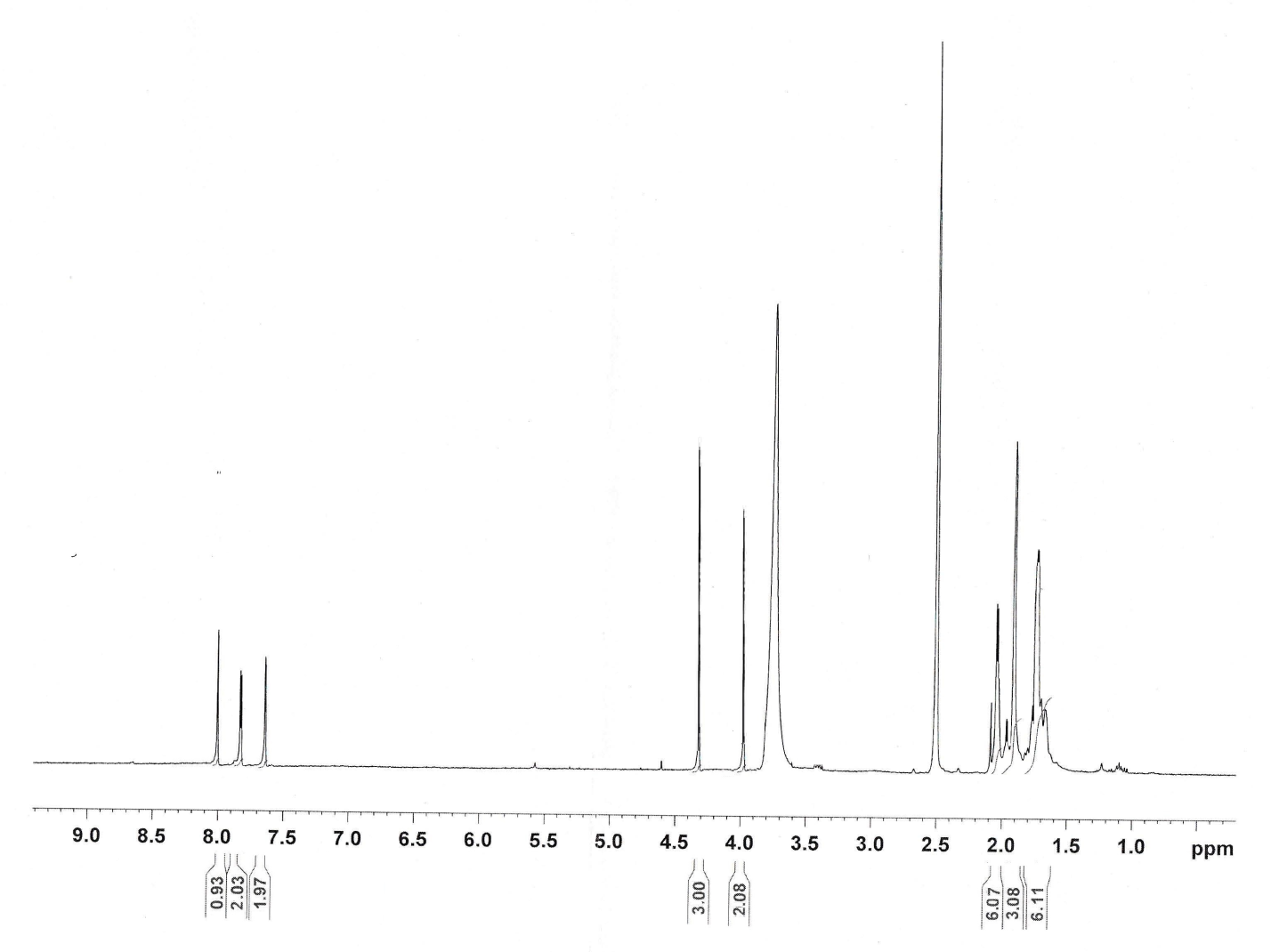


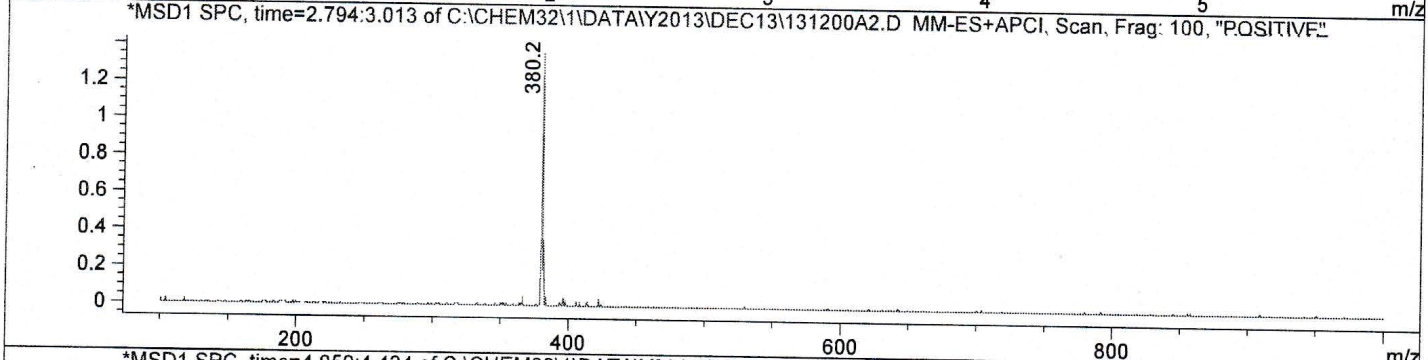

3e) 6-(adamantan-1-yl)-2-(furan-2-yl)imidazo[2,1-b][1,3,4]thiadiazole: 1HNMR (400 MHz, CDCl3) 8.1 (d, 1H), 8.00 (s, 1H), 7.4 (d, 2H), 6.8 (t, 1H), 2.1(m,6H),1.9(m, 3H), 1.7(m,6H); LCMS (MM:ES+APCI) 326.3(M+H)^+^ ; Anal.Calcd for C_18_H_19_N_3_OS : C 66.43; H 5.88; N 12.91. Found: C, 65.83; H, 6.05; N, 13,44.


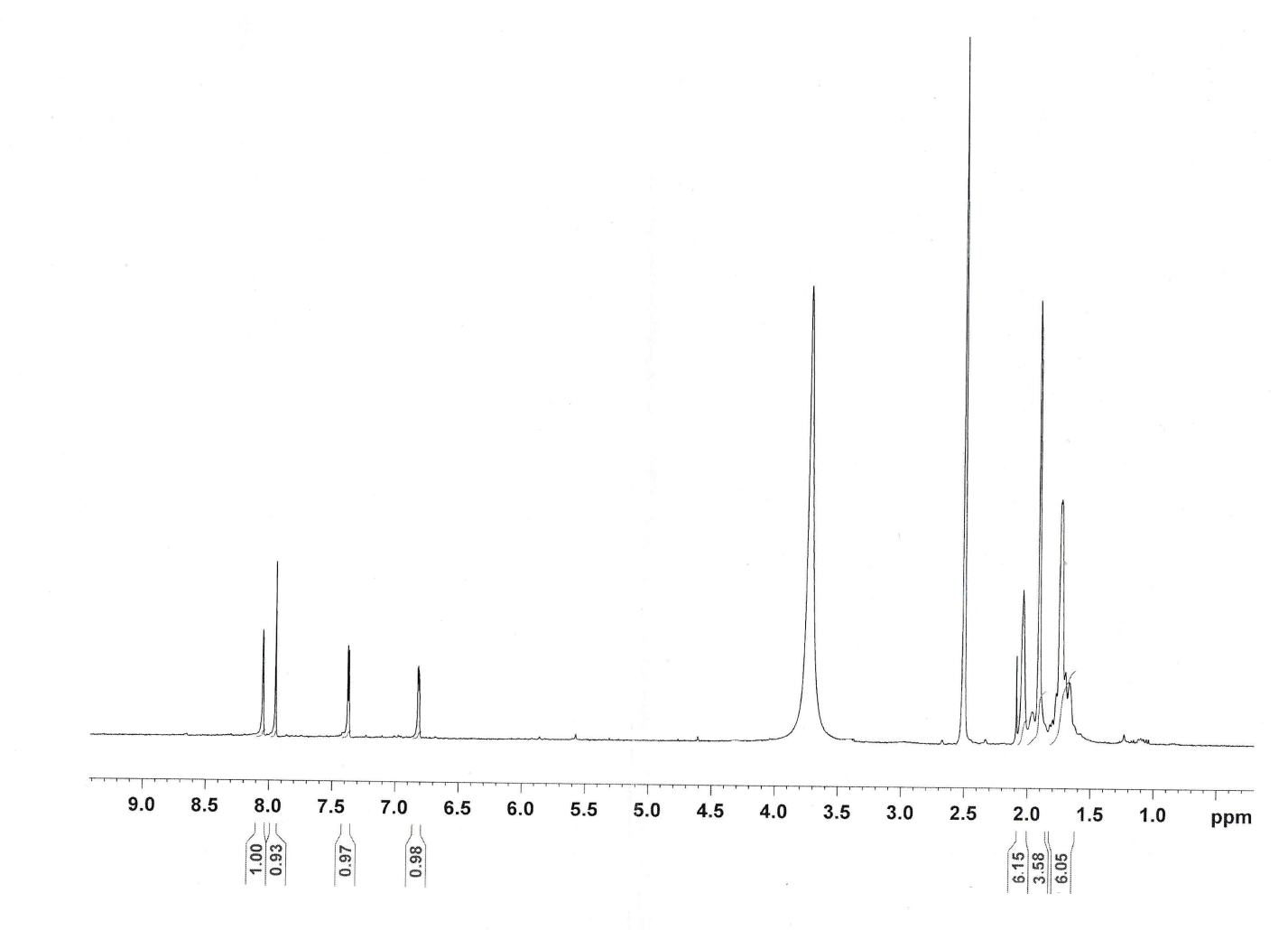


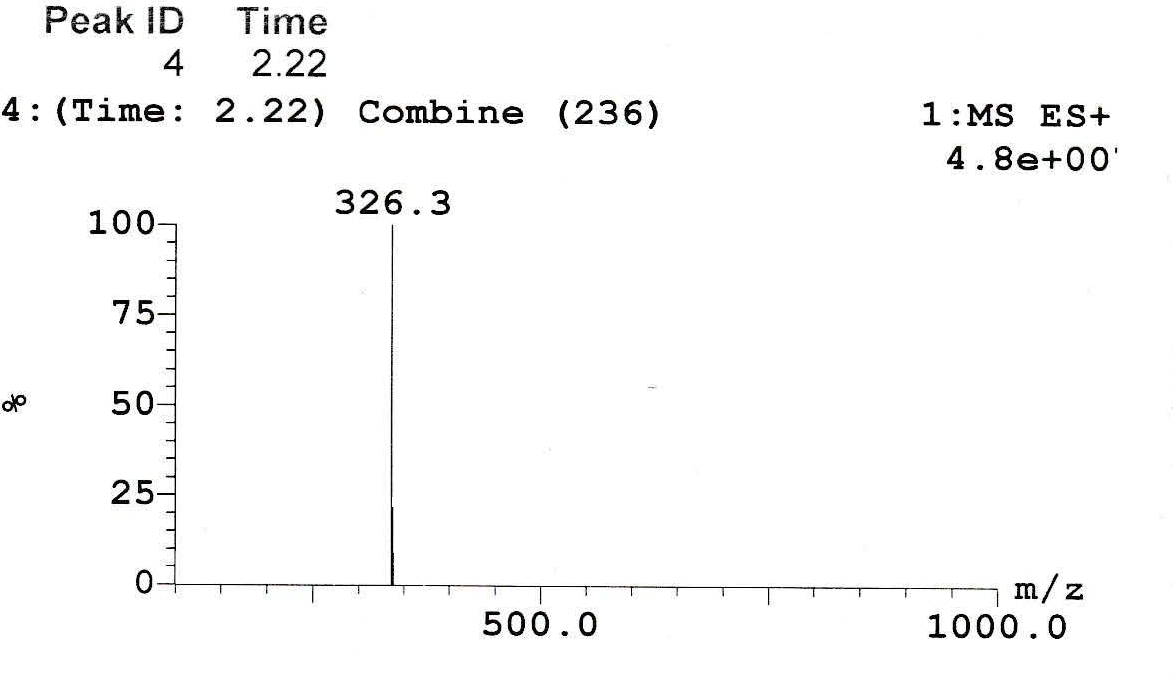

3f) 6-(adamantan-1-yl)-2-(4-methoxyphenyl)imidazo[2,1-b][1,3,4]thiadiazole: 1HNMR (400 MHz, CDCl3) 8.2 (d, J = 8.4 Hz, 1H), 8.00 (s, 1H), 7.8 (d, 2H), 4.4 (s, 3H), 2.1(m,6H), 1.9-1.7(m,9H); LCMS (MM:ES+APCI) 366.6(M+H)^+^ ; Anal.Calcd for C_21_H_23_N_3_OS : C 69.01; H 6.34; N 11.50. Found: C, 68.33; H, 5.89; N,11.10


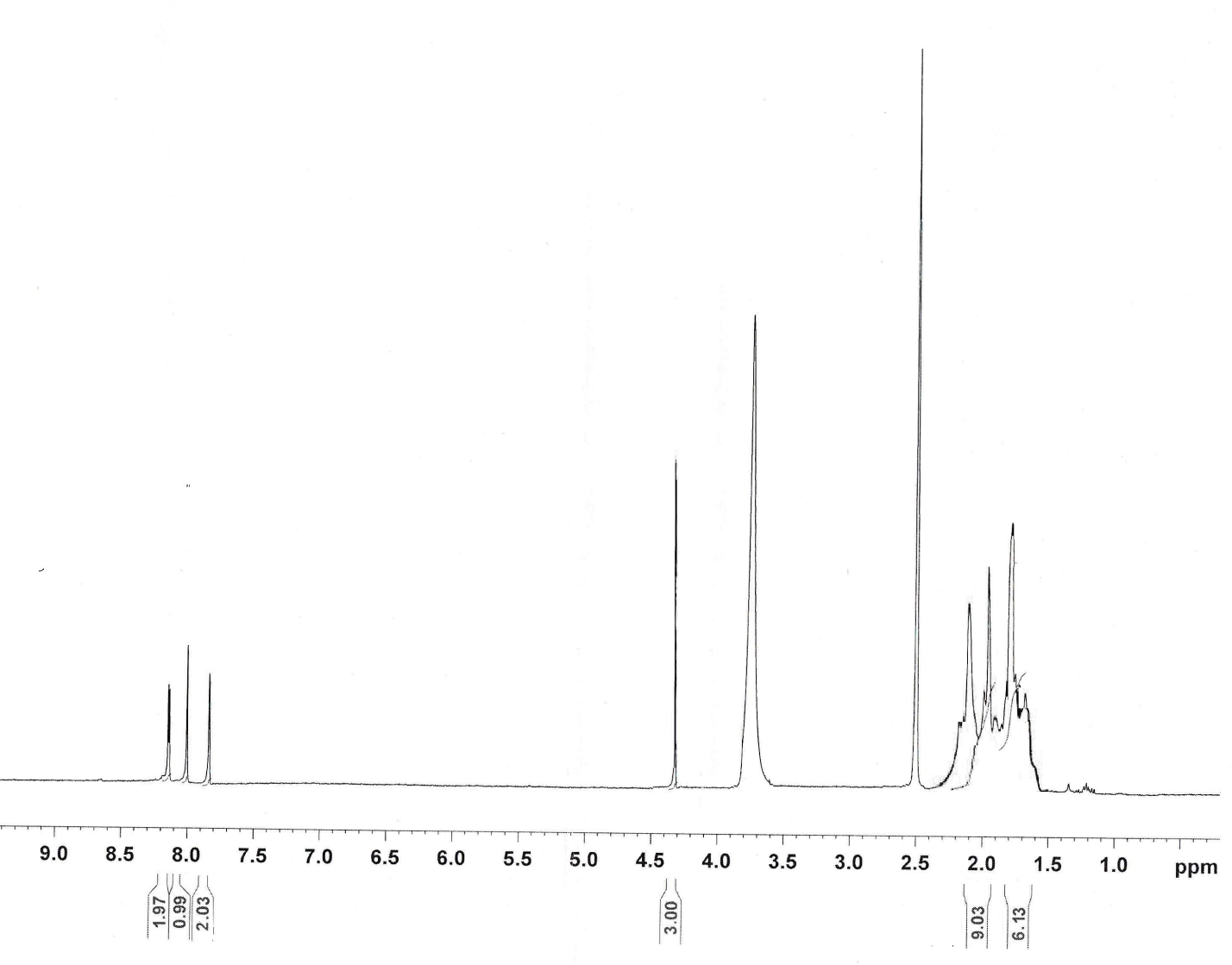


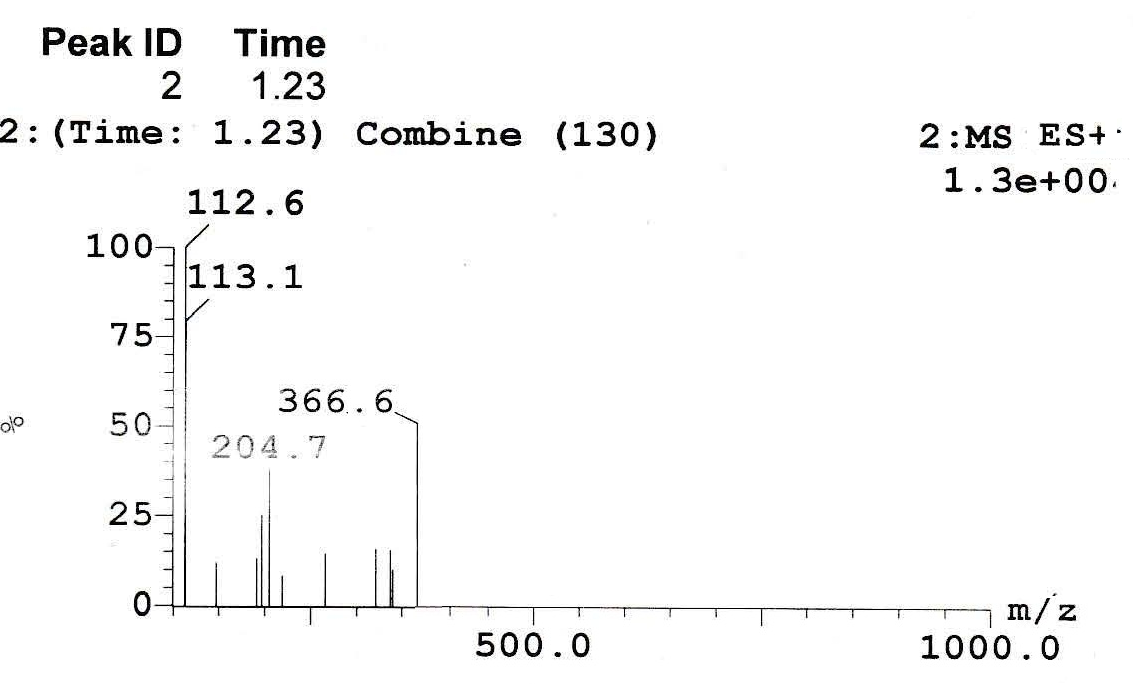

3g) 6-(adamantan-1-yl)-2-(4-bromophenyl)imidazo[2,1-b][1,3,4]thiadiazole: 1HNMR (400 MHz, CDCl3) 8.3 (d, 2H), 8.1 (d, 2H), 8.0 (s, 1H), 2.0(m,6H),1.8(m, 3H), 1.6(m,6H); LCMS (MM:ES+APCI) 415.1(M+H)^+^ ; Anal.Calcd for C_20_H_20_BrN_3_S : C 57.97; H 4.87; N 10.14. Found: C, 57.01; H, 4,22; N, 9.56.


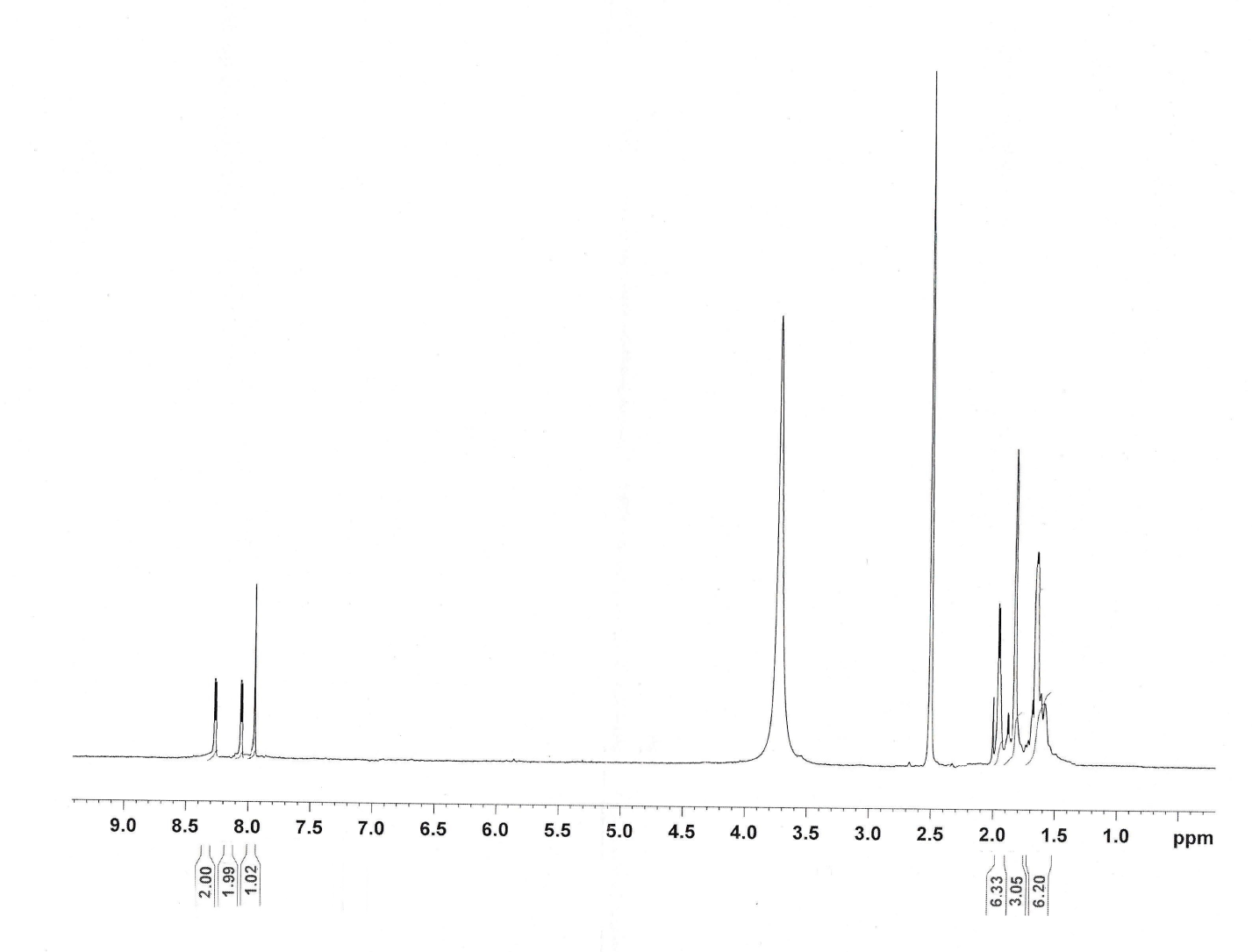


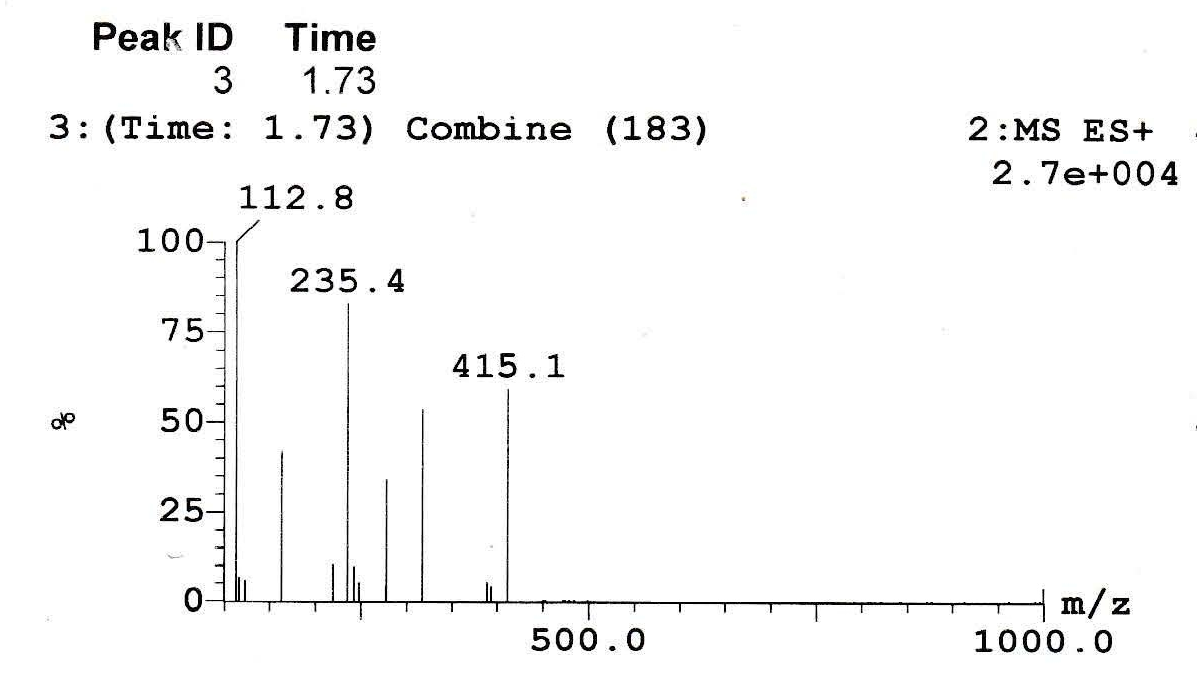

3h) 6-(adamantan-1-yl)-2-cyclohexylimidazo[2,1-b][1,3,4]thiadiazole: 1HNMR (400 MHz, CDCl3) 8.0 (s, 1H), 2.7 (m, 2H), 2.1- 1.8 (m, 15H), 1.7 (m, 6H), 1.5-1.4(m, 3H); LCMS (MM:ES+APCI) 342.3(M+H)^+^ ; Anal.Calcd for C_20_H_27_N_3_S : C 70.34; H 7.97; N 12.30. Found: C, 70.87; H, 8.54; N, 11.67.


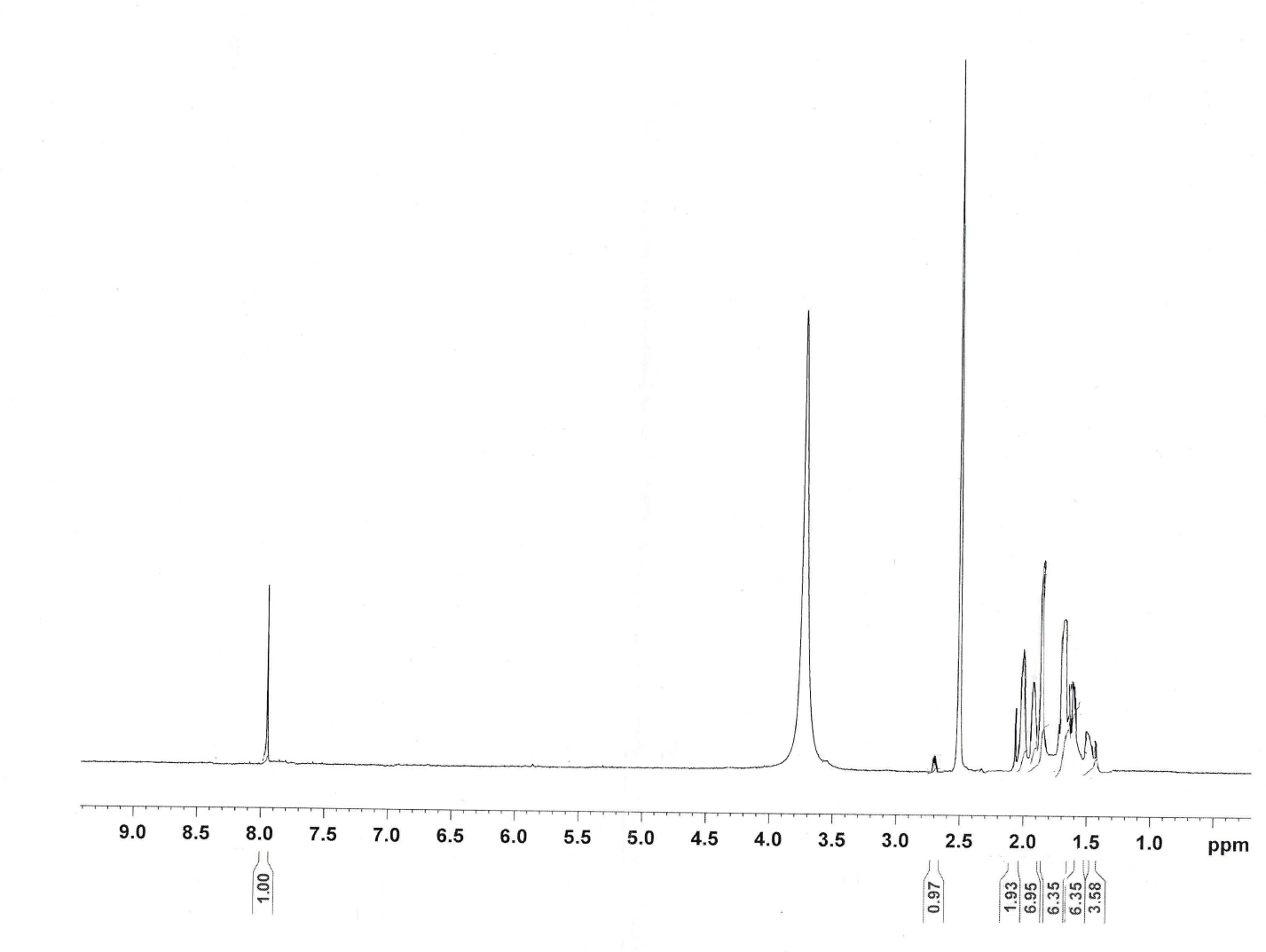


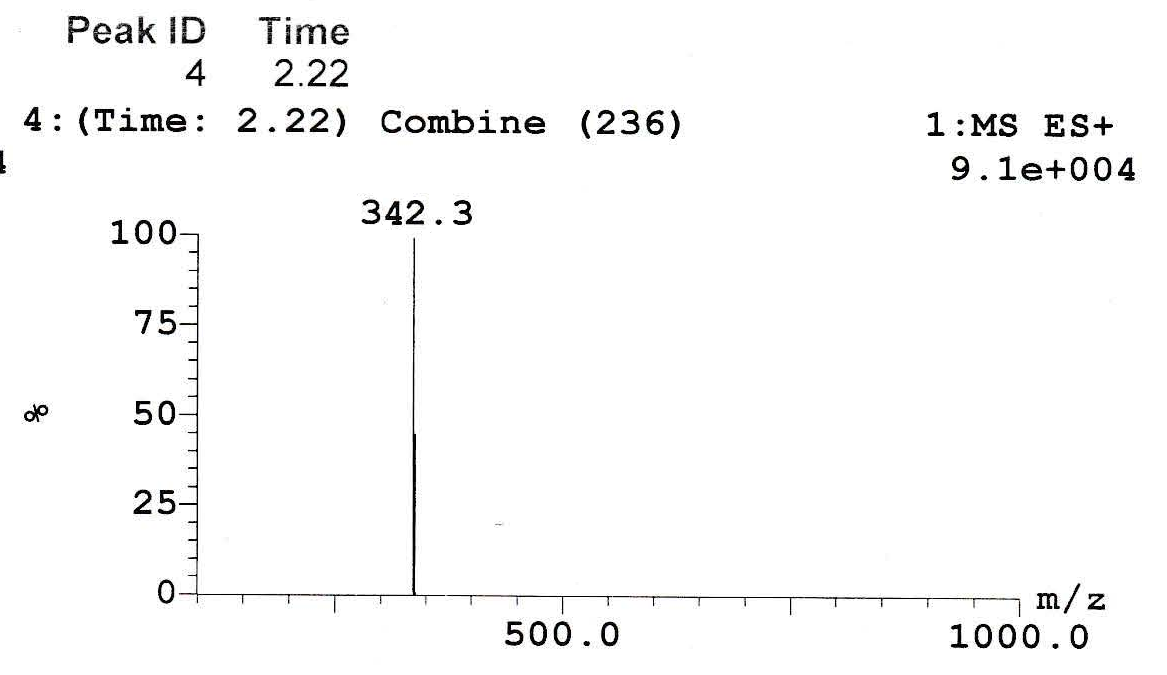

3i) 6-(adamantan-1-yl)-2-(trifluoromethyl)imidazo[2,1-b][1,3,4]thiadiazole: 1HNMR (400 MHz, CDCl3) 8.0 (s, 1H), 2.1(m,6H),1.9(m, 3H), 1.7(m,6H); LCMS (MM:ES+APCI) 328.4(M+H)^+^ ; Anal.Calcd for C_15_H_16_F_3_N_3_S : C 55.03; H 4.93; N 12.84. Found: C, 55.37; H, 4.44; N, 12.13.


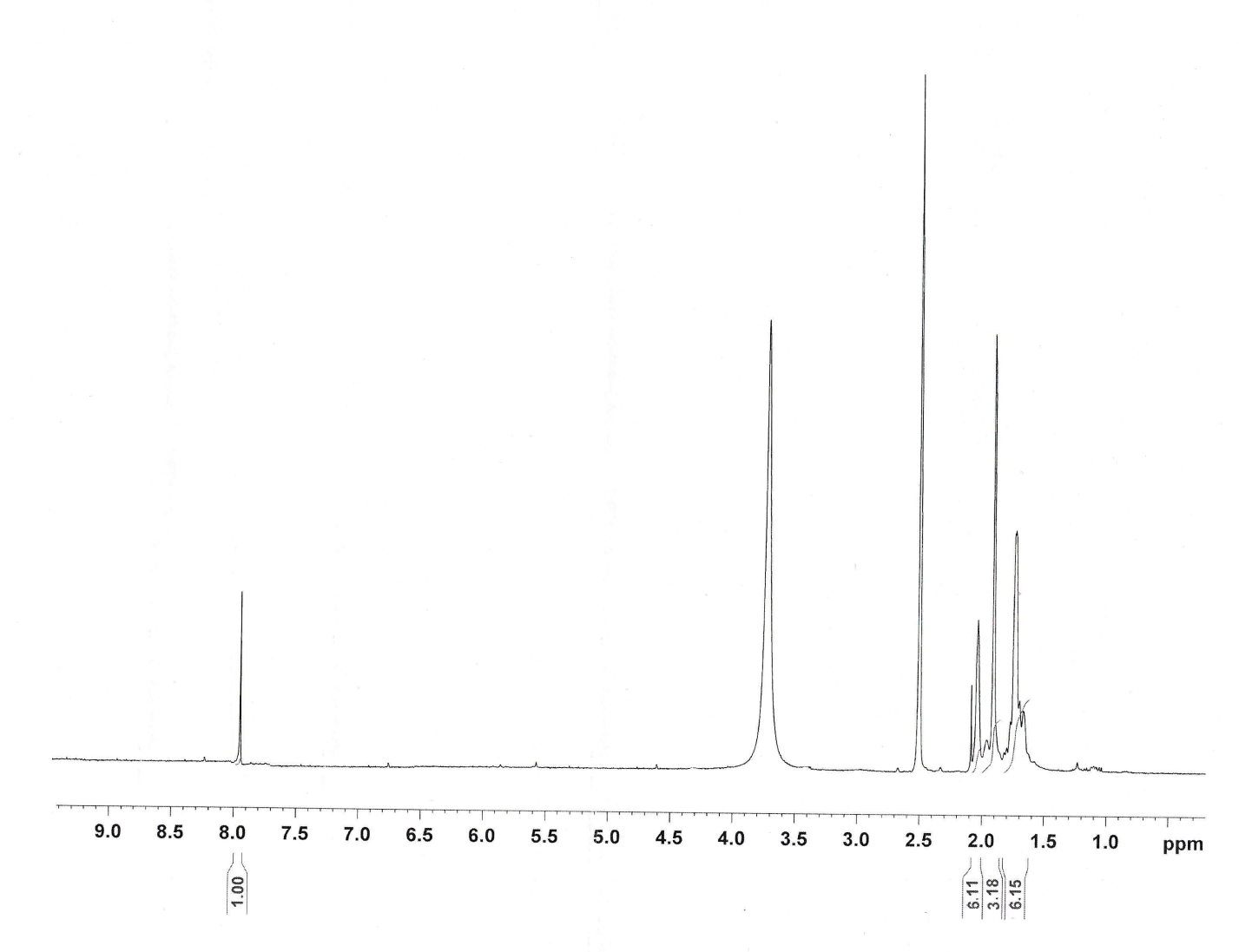


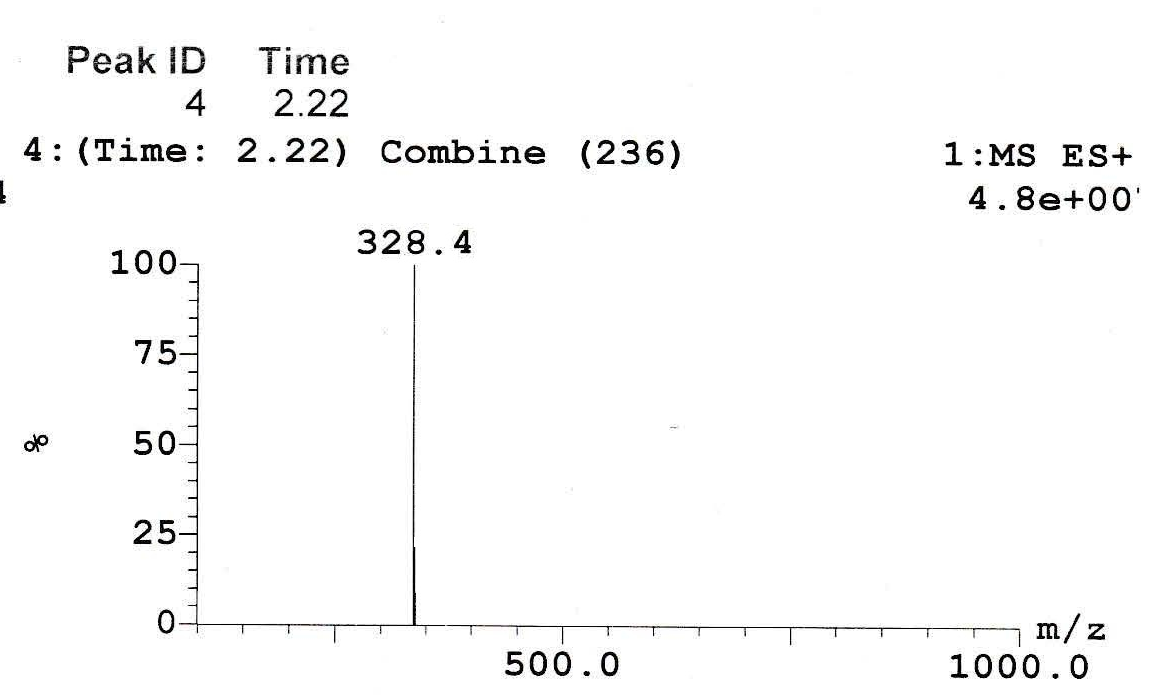

3j) 6-(adamantan-1-yl)-2-methylimidazo[2,1-b][1,3,4]thiadiazole 1HNMR (400 MHz, CDCl3) 8.0 (s, 1H), 2.8(s, 3H), 2.1(m,6H), 1.9(m, 3H), 1.7(m,6H); LCMS (MM:ES+APCI) 274.4(M+H)^+^ ; Anal.Calcd for C_18_H_14_FNO : C 65.90; H 7.00; N 15.37. Found: C, 66.09; H, 7.65; N, 15.89.


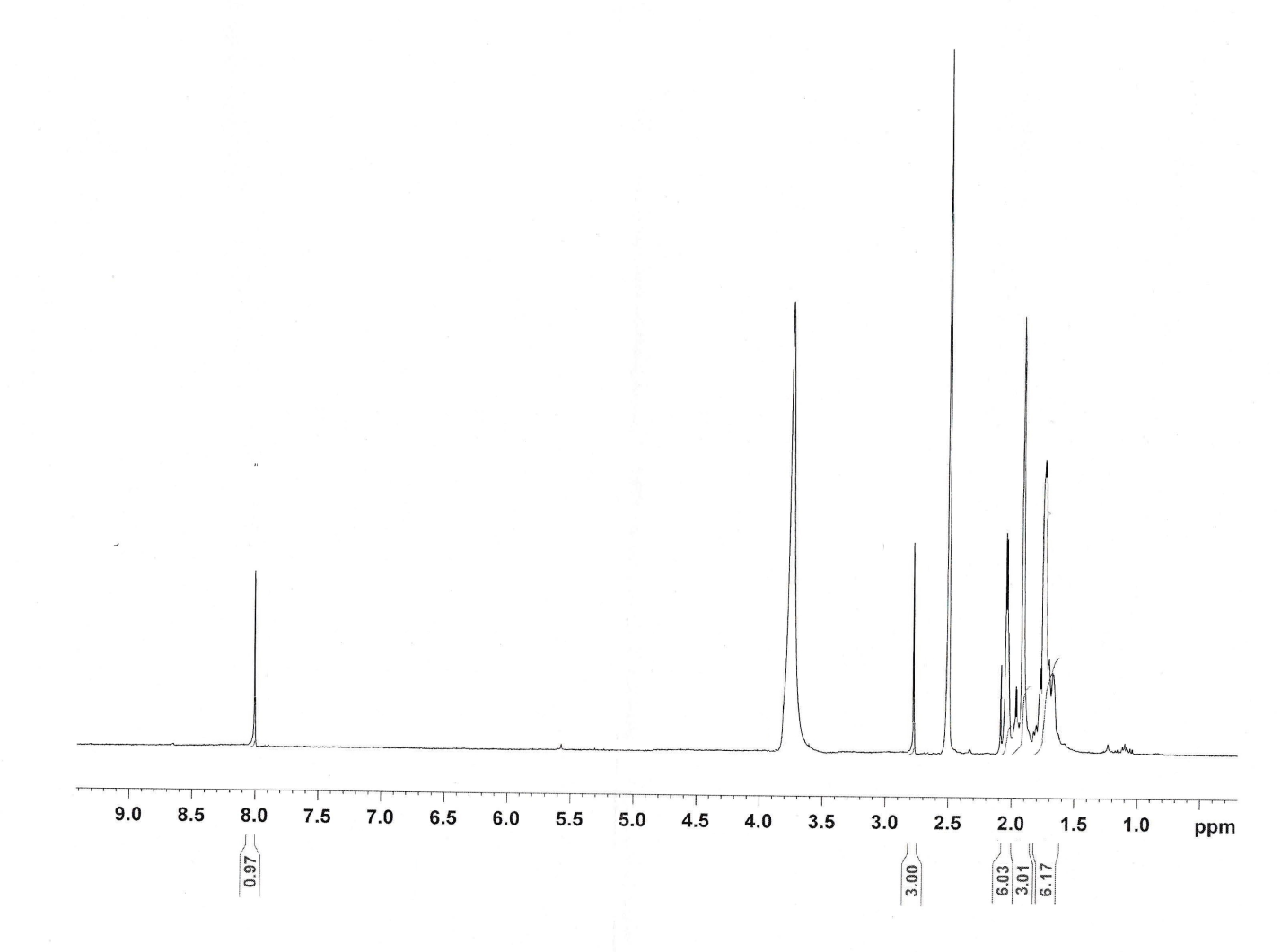


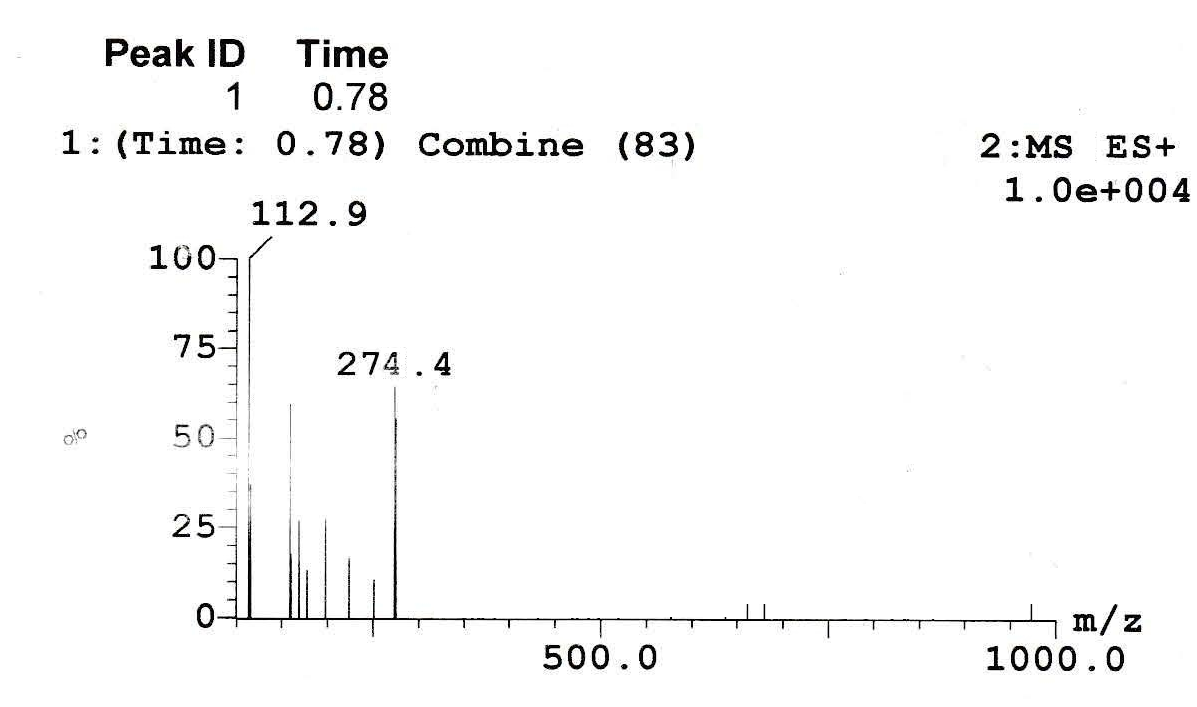

Supplement: S2 Data — (DOCX) [file pone.0139798.s002.docx]
